# A pragmatic cluster randomised controlled trial of a Diabetes REcall And Management system: the DREAM trial

**DOI:** 10.1186/1748-5908-2-6

**Published:** 2007-02-16

**Authors:** Martin P Eccles, Paula M Whitty, Chris Speed, Ian N Steen, Alessandra Vanoli, Gillian C Hawthorne, Jeremy M Grimshaw, Linda J Wood, David McDowell

**Affiliations:** 1 Centre for Health Services Research, University of Newcastle, Newcastle upon Tyne, UK; 2 Diabetes Centre, Newcastle Primary Care Trust, Newcastle upon Tyne, UK; 3 Clinical Epidemiology Program, Ottawa Health Research Institute, and Department of Medicine, University of Ottawa, Ottawa, Canada; 4 Northern and Yorkshire Regional Office, Diabetes UK, Darlington, UK; 5 c/o ProWellness UK Ltd, Centre 500, 500 Chiswick High Road, London W4 5RG, UK

## Abstract

**Background:**

Following the introduction of a computerised diabetes register in part of the northeast of England, care initially improved but then plateaued. We therefore enhanced the existing diabetes register to address these problems. The aim of the trial was to evaluate the effectiveness and efficiency of an area wide 'extended,' computerised diabetes register incorporating a full structured recall and management system, including individualised patient management prompts to primary care clinicians based on locally-adapted, evidence-based guidelines.

**Methods:**

The study design was a pragmatic, cluster randomised controlled trial, with the general practice as the unit of randomisation. Set in 58 general practices in three Primary Care Trusts in the northeast of England, the study outcomes were the clinical process and outcome variables held on the diabetes register, patient-reported outcomes, and service and patient costs. The effect of the intervention was estimated using generalised linear models with an appropriate error structure. To allow for the clustering of patients within practices, population averaged models were estimated using generalized estimating equations.

**Results:**

Patients in intervention practices were more likely to have at least one diabetes appointment recorded (OR 2.00, 95% CI 1.02, 3.91), to have a recording of a foot check (OR 1.87, 95% CI 1.09, 3.21), have a recording of receiving dietary advice (OR 2.77, 95% CI 1.22, 6.29), and have a recording of blood pressure (BP) (OR 2.14, 95% CI 1.06, 4.36). There was no difference in mean HbA1c or BP levels, but the mean cholesterol level in patients from intervention practices was significantly lower (-0.15 mmol/l, 95% CI -0.25, -0.06). There were no differences in patient-reported outcomes or in patient-reported use of drugs, or uptake of health services. The average cost per patient was not significantly different between the intervention and control groups. Costs incurred in administering the system at the register and in general practice were in addition to these.

**Conclusion:**

This study has shown benefits from an area-wide, computerised diabetes register incorporating a full structured recall and individualised patient management system. However, these benefits were achieved at a cost. In future, these costs may fall as electronic data exchange becomes a reliable reality.

**Trial registration**: International Standard Randomised Controlled Trial Number (ISRCTN) Register, ISRCTN32042030.

## Background

There is broad, international agreement over what constitutes high-quality health care for people with diabetes [1,2]. In the United Kingdom (UK), this has been captured in a National Service Framework for people with diabetes [3]. At the time of setting up the Diabetes REcall And Management system (DREAM) trial, computerised central recall systems for patients and their family doctors had been supported by the evidence from a 1999 systematic review [4]. However, the evidence base on which these conclusions were based was limited to that from patient- rather than practice-randomised trials, in selected practice samples, and without economic evaluation. Thus the effectiveness of an area-wide, patient-focussed, structured recall and management system (in terms of process of care, patient outcome, and economic impact) remained unknown. A recent systematic review of quality improvement interventions to improve the quality of care in patients with diabetes showed that a range of different interventions resulted in small to modest improvements in glycemic control and in provider adherence to optimal care [5]. Across 59 studies (only five from the UK), they reported a median absolute reduction in serum HbA_1c _of 0.48 and a median absolute increase in provider adherence of 4.9%. However, they also identified important methodological concerns, with larger studies and randomised studies showing smaller benefits than smaller or non-randomised ones, which strongly suggest the presence of publication bias. Studies in the highest quartile of sample size reported a median reduction in serum HbA_1c _of only 0.10%.

Within their taxonomy of interventions the categories of "provider reminders" and "audit and feedback" most closely approximate to the intervention in this study. Across 14 trials examining one or both of these interventions, they found median improvements in provider adherence of between 4% and 8%, and improvements in HbA1c of around 0.1%[5]. They also examined 38 comparisons involving some form of clinical information system to deliver the intervention, finding no incremental benefit for any particular informatics function (i.e., decision support, auditing clinical performance, reminder systems), over and above delivering the function without an informatics system.

Following the introduction of a computerised diabetes management system in three (then) Primary Care Group areas, in the northeast of England, care initially improved but then plateaued, a phenomenon also reported by others [6,7]. At the point this assessment of care was performed, the measures of care were restricted to documenting the performance of various actions (e.g. measurement of BP) rather than documenting the values. We postulated that the platueauing was due to clinicians failing to deliver appropriate clinical interventions due to a lack of coordination (i.e., patients being lost to follow-up), and either a lack of awareness of appropriate care or forgetting to deliver all that was required when patients were seen. Therefore, we developed the diabetes register system to address these problems.

This study aimed to evaluate, within a pragmatic, cluster randomised controlled trial design, the effectiveness and efficiency of an area-wide, 'extended' computerised diabetes register incorporating a full-structured recall and management system, actively involving patients, and including individualised patient-management prompts to primary care clinicians based on locally-adapted, evidence-based guidelines.

## Methods

The study methods described here are reported in detail elsewhere [8].

### Study general practices and registers

The study general practices were those in three Primary Care Trusts (PCTs) served by two district hospital-based diabetes registers, both using the same register software. When the study was designed, it was based in three PCTs (all agreed to participate in the study) served by a single register. However, the withdrawal of one of these PCTs necessitated the recruitment of a replacement PCT served by a second register. Several factors led to the withdrawal of this PCT. Despite our having appropriate administrative approval, when the trial began it became apparent that the administrative authority did not have the cooperation necessary for all of the GPs to participate in the trial. Consequently we had to enrol individual practices directly (rather than via the PCT), which resulted in fewer practices enrolling and our being at risk of not achieving our required sample size. We recruited a further PCT to address this problem, however, the original PCT then suspended involvement with the diabetes register and their practices had to be excluded from the study. This was a deviation from the published protocol.

### Study patients

Study patients were those people with type 2 diabetes appearing on the registers, aged over 35 years and receiving diabetes care exclusively from study general practices or shared between study general practices (GPs) and hospital. At the time of the study, approximately 20% of patients received both GP and specialist care, though there was no formal shared-care scheme in operation in the PCTs studied.

### Study design, outcomes and power

The study was a pragmatic two-arm cluster randomised controlled trial with the general practice as the unit of randomisation. Randomisation was performed using electronically-generated random numbers by the study statistician and was stratified by PCT and practice size.

The study outcomes were: the clinical process and outcome variables held on the diabetes registers; patient reported outcomes (the SF36 health status profile [9-11], the Newcastle Diabetes Symptoms Questionnaire [12], and the Diabetes Clinic Satisfaction Questionnaire [13]); and service and patient costs. Patients have been shown to be able to report cost data reliably [14].

As this was a quality of care study interested in a range of measures of care, it was important to use as study outcomes the routinely available process and outcome measures on which clinicians alter patients' care. Our power calculation was based on indicative process and outcome variables. The intra-cluster correlation coefficient (ICC) for measures of process calculated from local data was 0.14, whether a blood pressure measurement or an HbA1c measurement has been recorded in a 12-month period. Therefore, to detect a difference of 15% (42.5% v 57.5%) in a binary variable with 80% power, assuming a significance level of 5%, required 60 practices each contributing 30 patients [15]. The sample size for the outcome of care variables was based on the SF-36. Previous work had shown that where this type of intervention produces an effect, it was likely to produce an effect size of approximately 0.25 in such measures [16] – and that the ICCs for such measures would be approximately 0.07 [17]. A final sample of 27 patients from each of 61 practices would give 85% power to detect an effect size of 0.25, assuming a significance level of 5%. Assuming a response rate of 70%, the starting sample size was 2379 patients (approximately 39 patients per practice).

### Data collection

We collected process data for the 12 months preceding the start of the intervention and for the 15 months of the intervention period (1^st ^April 2002 to 30^th ^June 2003). All data were extracted from the registers at the end of the intervention period. Prescription data were similarly collected, but, because of problems reliably determining the date of initiation of prescriptions, we collected drug data back to the point at which a study patient first appeared on the register. We gathered data on patient reported outcomes by postal questionnaire at the end of the intervention period. Questions on the costs incurred by patients were developed by the study health economist and were included in the questionnaire. These questions included the self-reported use of medication. Non-responders to the initial posting received a reminder letter after two weeks; non-responders to this received a *second *reminder letter and a copy of the questionnaire after a further two weeks.

We gathered information on workload and other resource impacts of the intervention in general practice, with a semi-structured telephone interview survey of key informants within a random sample of 10 intervention and 12 control practices. Similar information on the impact on the registers was collected by the register staff, logging time spent on intervention-related activities.

### Analysis

The following analytic strategies were adopted. For the process of care and intermediate outcome variables collected directly from the register, the dependent variable took the form of an observation for an individual patient in the period after implementation of the intervention. We had data on these variables both before and after the intervention, and, for each variable considered, the post intervention measure was specified as the dependent variable and the corresponding pre-intervention measure was specified as a covariate. The effect of the intervention was estimated using generalised linear models with an appropriate error structure (binomial for binary data, normal for continuous data, and negative binomial for count data) and link function (logit for binary data, identity for continuous data, and log for count data). To allow for the clustering of patients within practices, population averaged models were estimated using generalized estimating equations (GEEs). Baseline variables (pre-intervention data) were included in the model as a covariate.

Examination of the drug therapy data suggested that the variable that was recorded most reliably on the register was the date that the medication was started. In general, patients who started on a particular medication prior to the intervention period also were taking that medication during the intervention period. For each type of medication, the total number of patients prescribed that medication in each practice was determined. This variable was analysed using negative binomial regression with the total number of relevant patients in the practice included as an exposure variable; the number of patients prescribed that medication prior to the intervention was included as a covariate.

Questionnaire data were only available following the intervention. Patient-reported outcome measures were analysed using population averaged models as described for the process data above, except that, as we had no pre-intervention measure, no adjustment for differences at baseline was possible, thus no baseline covariate was included in the model. Patient reported medication data were analysed for the register medication data, except that, again, there were no baseline data to include as a covariate.

In addition to the above analyses that were pre-specified, because of large systematic differences between the two registers that became apparent once the data had been collected, a further model was fitted which included a register effect. This was not pre-specified, but the differences were so large it was felt that it would be inappropriate to ignore them during the main analysis. All analyses were undertaken using Stata version 8.

The economic evaluation adopted a 'cost consequences' approach [18]. All costs were expressed in 2002/2003 values. Two main sources were used to assign costs to health care resources [19,20] supplemented when necessary with unit cost data from other official sources [21] and local surveys. Drug costs were taken from the British National Formulary [22]. Patients reported on the use of NHS (National Health Service) services, medications, travel costs, costs for the purchase of special items, private treatments/consultations and time off work, sick leave and related pay loss, as well as time off work and related pay loss to their companions over a twelve-month period. No discounting was applicable. A simplifying assumption was made that the use of all costs and resources occurred at the beginning of this period.

### Intervention

The development and implementation of the intervention have been described in detail elsewhere [23]. In summary the pre-existing diabetes register functioned as a central register of patients with diabetes. A structured dataset was completed on paper forms and returned to the central register; the hospital laboratory provided a monthly download of laboratory test results (e.g. HbA1c) for patients on the register. From this data both patient-specific and aggregated data were provided annually to patients and clinicians. The pre-existing system was passive, in that it did not request data for patients, rather it summarised the data it received. We postulated that the platueauing of performance that had been documented was due to clinicians failing to deliver appropriate clinical interventions due to a lack of co-ordination (patients being lost to follow up) and either a lack of awareness of appropriate care, or forgetting to deliver all that was required when patients were seen.

In the enhanced structured and recall management system, a 'circle of information exchange' was established between the participating general practices and the database. The central database system identified when patients were due for review and generated a letter to the patients asking them to make an appointment for a review consultation. The rules for generation of review letters were adapted for each PCT area. In one PCT, the system acted as a prompting system for annual review, and patients were identified 11 months after their last diabetes appointment. In the other two PCTs, patients who had missed annual reviews were identified by searching for patients who had not had a diabetes appointment for 14 months or more. At the same time, the central database generated a letter to the practice stating that the patient should be making a review appointment in the near future. The letter to the practice included a 'structured management sheet' (to be held in the patient's record) to capture an agreed minimum data set that would be collected during the consultation. This management sheet also contained relevant prompts tailored to a patient's known clinical or biochemical values, derived from locally adapted, national evidence-based guidelines [see Additional file 1].

When the patient was seen in the practice, the primary care professional (often the practice nurse) completed the management sheet and returned a copy for entry into the central register within a designated period of time. This circle of information was broken if the patient did not visit the general practice as planned or the general practice did not return the management sheet to the central register. If this happened, the central register would print reminder letters and further structured management sheets at the next routine database search by the diabetes register facilitator, which occurred at least weekly.

In addition to this cycle based on annual reviews, routine ongoing structured management sheets were produced every time a patient in an intervention practice was identified by the diabetes register facilitator on the register database. For example, when data were inputted on the database for any reason, the system would print a structured management sheet updated for any new data and relevant management prompts, and this would be sent to the relevant practice.

The trial intervention ran for 15 months, commencing on 1 April 2002 and ending on 30 June 2003. The letters to patients inviting them for annual review commenced in October 2002 – delayed to overcome concerns about the accuracy of patient details on the database up to this point. The enhanced system also was capable of producing patient letters to accompany routine ongoing structured management sheets for practices, but because of difficulties operating this element of the software it was not possible to run this feature during the lifetime of the trial. This was a deviation from the published protocol.

### Ethics

The study was approved by the South Tyneside, Southwest Durham, Hartlepool, and North Tees Local Research Ethics Committees (LRECs).

## Results

**Figure 1:**
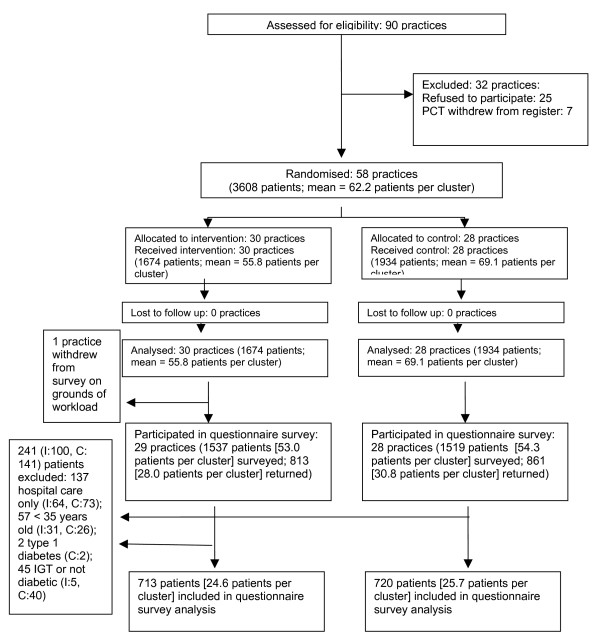
Flow of clusters and individual participants through each stage of recruitment, randomisation and analysis.

Figure 1 shows the number of practices and patients at each stage of the study. It was not possible to provide the number of patients within the 90 practices assessed for eligibility, as we did not have ethical approval to access data on the patient inclusion criteria for practices that had not agreed to participate in the study. As a condition of ethical approval in one of the PCTs, individual opt-out consent had to be sought from patients whose practices had agreed to participate: 477 out of 4577 (10.4%) patients invited to participate opted out of the trial. [The considerably higher number of patients written to as compared to the number of patients included in the trial reflected the need to get permission before being able to access the diabetes register – and then apply the inclusion criteria.] Table 1 shows the baseline characteristics of control and intervention practices and patients. None of the differences in these variables between the intervention and control group are statistically significant. Unfortunately, we were unable to compare the clinical characteristics of respondents and non-respondents to the patient survey, as we were subject to the requirement of the ethics/research governance organisations that we should not hold any patient-identifiable data within our academic institution. We were supplied with a list of names and addresses of patients to whom we could send out a patient survey, but were not allowed access to link that information with individual patient records on the registers.

**Table 1:** Baseline characteristics of control and intervention practices and patients.

	**Control group (n 28)**	**Intervention group (n 30) **
**Practice factors at baseline**		
Number of partners:		
Single-handed	9	10
2 to 4 partners	15	16
5 to 7 partners	4	2
>7 partners	0	2
Number of practices with a Practice nurse	28	30
Patient factors at baseline*		
Number	1934	1674
Mean (sd) age (years)	66.6 (11.3)	65.7 (11.8)
No (%) men	1001 (52%)	901 (54%)
No (%) on diet only	947 (49.1%)	980 (59.0%)
No (%) on oral hypoglycaemics (sulphonylureas, biguanides, thiazols) but not on insulin	923 (47.9%)	628 (37.8%)
No (%) on insulin	57 (3.0%)	54 (3.2%)

* Data from Diabetes Register. (No data were available from the diabetes register on ethnicity, however, the proportion of people from ethnic minority groups in the study PCTs is very low.^21^)

The findings from analysis of the process of care clinical variables and drug data from the register-derived dataset are shown in Table 2. This analysis is adjusted for differences at baseline and a systematic difference between registers. Analyses allowing for baseline data *only *and register effect *only *are presented alongside this analysis in Additional file 2 [see Additional file 2]. Nineteen subjects (7 in control group, 12 in intervention group) had no valid date in their medication record and were excluded from the medication analysis. With the exception of serum creatinine, a variable that we anticipated that the intervention would not influence, all of the variables measured showed a direction of effect in favour of the intervention. For 10 of the 26 variables measured, this difference achieved statistical significance.

**Table 2:** Adjusted register-derived process and clinical outcome data results for intervention and control groups. Odds ratios are estimates of the difference between intervention and control practices at follow-up, adjusting for differences at baseline and a systematic difference between registers.

	**Control Practices**		**Intervention Practices**		
**Measures**	**Baseline**	**Follow-up**	**Baseline**	**Follow-up**	

**Attendance**					**Odds Ratio (95% CI)**
Proportion of patients with at least one appointment	73.4%	67.7%	74.3%	81.7%	2.00* (1.02, 3.91)
Mean number of appointments	1.23	1.35	1.29	2.02	Relative Risk 1.26 (0.87, 1.81)
**Process of care**					
Fundoscopy recorded	49.5%	50.5%	43.1%	60.6%	1.45 (0.88, 2.40)
Feet examination recorded	46.1%	48.8%	48.0%	67.3%	1.87*(1.09, 3.21)
Dietary advice recorded	19.9%	29.2%	25.3%	46.3%	2.77*(1.22, 6.29)
Smoking status recorded	34.2%	48.0%	36.9%	66.0%	2.43*(1.18, 5.00)
Was subject a smoker?	19.3%	19.6%	20.7%	21.4%	0.72 (0.38, 1.37)
BP recorded	59.3%	48.3%	55.3%	71.4%	2.14*(1.06, 4.36)
HbA1c recorded	64.0%	66.0%	60.9%	79.0%	1.58 (0.81, 3.08)
Cholesterol recorded	57.0%	61.1%	53.3%	78.0%	1.66 (0.89, 3.12)
Creatinine recorded	48.0%	60.4%	53.0%	73.4%	1.36 (0.72, 2.52)
Albumin:creatinine ratio recorded	26.8%	29.7%	30.2%	40.4%	1.60(0.98, 2.60)
**Clinical **					**Difference**
Mean most recent systolic blood pressure	144.5	144.6	145.8	144.2	-1.56 (-4.54, 1.42)
Mean most recent diastolic blood pressure	80.2	78.1	79.2	77.8	-0.40 (-1.78, 0.97)
Mean most recent HbA1c^#^	7.56	7.35	7.75	7.32	-0.04 (-0.18, 0.10)
Mean most recent cholesterol^#^	5.27	5.06	5.23	4.94	-0.15**(-0.25, -0.06)
Mean most recent creatinine^#^	93.1	96.1	91.8	95.7	0.21 (-1.27, 1.70)
Mean most recent albumin:creatinine ratio^#^	8.99	8.45	8.48	8.05	-1.6 (-4.4, 1.2)
**Diabetes medication**					**Relative risk (95% CI)**
Biguanide, Sulphonylurea or Thiazol	944 (49.0%)	1128 (58.5%)	646 (38.9%)	923 (55.5%)	1.06 (0.94, 1.19)
Metformin	424 (22.0%)	573 (29.7%))	343 (20.6%	530 (31.9)	1.07 (0.81, 1.41)
Insulin	57 (3.0%)	75 (3.9%)	54 (3.2%)	75 (4.5%)	1.15 (0.83, 1.58)
**Cardiovascular risk factor drugs**					
Aspirin	10 (0.5%)	164 (8.5%)	34 (2.0%)	308 (18.5%)	2.08* (1.00, 4.32)
Ace Inhibitor	17 (0.9%)	103 (5.3%)	31 (1.9%)	185 (11.1%)	2.03* (1.08, 3.78)
ACE inhibitor or Angiotensin-II receptor antagonist	21 (1.1%)	109 (5.7%)	38 (2.3%)	192 (11.6%)	1.86* (1.03, 3.38)
Any antihypertensive	118 (6.1%)	274 (14.2%)	131 (7.9%)	415 (25.0%)	1.89*(1.16, 3.08)
Lipid-lowering	110 (5.7%)	290 (15.0%)	79 (4.8%)	418 (25.2%)	1.66 (0.99, 2.79)
**Any medication**	1674 (86.9%)	1838 (95.4%)	1283 (77.2%)	1549 (93.2%)	1.01 (0.94, 1.08)

*p < 0.05, **p < 0.01, *** p < 0.001

# Data downloaded into register database directly from hospital laboratory information system.

### Patient reported outcome data

We surveyed a random sample of 3056 patients, receiving usable responses from 1433. With 241 exclusions, this gave an overall response rate of 51% (number of eligible subjects who responded divided by the number of people sampled, minus those known to be ineligible) (Figure 1). There were no statistically significant differences in response rate between intervention and control group respondents, or on any sociodemographic variables. Analyses of the patient-reported medication data are summarised in Table 3. The differences between intervention and control groups were not statistically significant. There were no differences between the two registers, so the adjusted values differ little from the unadjusted ones. The patient-reported outcome data from the questionnaire survey are summarised in Table 4. The ICC for the diabetes symptom score was 0.03, and the ICCs for the SF 36 physical and mental health component scores were 0.03 and 0.02, respectively. There were no statistically significant differences in scores on any of the measures in Table 4, or on any of the items of the DCSQ.

**Table 3:** Self-reported medication data from the patient questionnaire survey.

**Drug category**	**% of subjects taking drug by group**		**Effect of intervention**		**Effect of intervention adjusted for a difference between registers**	
	
	**Control**	**Intervention**	**RR**	**95% CI**	**RR**	**95% CI**
***Diabetes medication***						
Diet alone	46.4	47.0	1.01	0.87, 1.18	1.02	0.89, 1.17
Any oral hypoglycaemic (biguanide, sulphonylurea or thiazolidinediones)	34.0	32.7	0.96	0.81, 1.14	0.96	0.81, 1.14
Sulphonylurea	19.7	18.6	0.94	0.75, 1.18	0.93	0.75, 1.16
Metformin	25.3	24.4	0.96	0.76, 1.22	0.97	0.77, 1.22
Insulin	24.4	26.8	1.10	0.84, 1.43	1.09	0.82, 1.37
***Cardiovascular disease and risk factor management***						
Any cardiovascular drug	49.6	45.9	0.92	0.84, 1.01	0.93	0.85, 1.01
Any anti-platelet drug	25.4	22.9	0.90	0.74, 1.10	0.90	0.75, 1.10
Aspirin	31.6	28.5	0.90	0.75, 1.08	0.90	0.75, 1.08
ACE inhibitor	25.0	22.1	0.89	0.75, 1.05	0.89	0.76, 1.05
Drugs primarily used as^a ^antihypertensives (including ACE/A-G inhibitors)	33.1	30.4	0.92	0.82, 1.03	0.92	0.83, 1.03
Any lipid-lowering	27.4	25.9	0.94	0.78, 1.14	0.95	0.78, 1.15
Statins	27.0	25.0	0.92	0.76, 1.12	0.93	0.77, 1.12
Fibrates	1.0	1.6	1.61	0.61, 4.27	1.59	0.60, 4.18

a. Categories of cardiovascular drugs can be prescribed for more than one purpose (e.g., beta-blockers may be used to treat hypertension but also treat angina), whereas individual drugs within categories (e.g., atenolol) may be better known to be used for a specific purpose. The drugs in this category were known to be used primarily as antihypertensives.

**Table 4:** Patient-reported outcomes

	**Raw data: mean scores (SD)**		**Estimated effect of intervention**	**Estimated effect of intervention adjusted for a difference between registers**
**Measure**	**Control**	**Intervention**	**Mean (95% CI)**	**Mean (95% CI)**

Diabetes symptom score	2.18 (0.71)	2.20 (0.71)	0.02 (-0.08, 0.12)	0.02 (-0.08, 0.12)
**SF-36**				
Physical function	48.8 (32.7)	48.9 (32.6)	-0.19 (-4.88, 4.50)	-0.17 (-4.87, 4.52)
Role physical	39.2 (43.8)	39.1 (44.5)	-0.40 (-6.85, 6.04)	-0.42 (-6.88, 6.03)
Bodily pain	52.9 (29.5)	52.8 (30.3)	-0.22 (-4.25, 3.82)	-0.18 (-4.24, 3.89)
General health	45.2 (23.1)	45.2 (23.7)	-0.09 (3.58, 3.41)	-0.05 (-3.52, 3.42)
Vitality	44.0 (23.0)	42.9 (23.8)	-1.53 (-4.52, 1.45)	-1.53 (-4.55, 1.48)
Social Function	66.4 (29.6)	64.0 (30.4)	-2.71 (-7.00, 1.56)	-2.71 (-7.03, 1.61)
Role emotional	54.1 (46.0)	52.9 (46.5)	-1.15 (-7.17, 4.87)	-1.22 (-7.21, 4.76)
Mental health	68.0 (20.4)	67.8 (20.3)	-0.13 (-3.14, 2.88)	-0.11 (-3.13, 2.91)
				
Physical health component score	30.1 (15.3)	29.7 (15.6)	-0.50 (-2.80, 1.80)	-0.50 (-2.82, 1.82)
Mental health component score	46.2 (11.8)	45.8 (12.1)	-0.35 (-1.96, 1.27)	-0.36 (-1.98, 1.26)

### Economic data

The economic data relating to service use and patient expenditure are summarised in Table 5, and were not significantly different between intervention and control groups. The intervention costs were: UK£11,443 for developing the local guidelines, UK£14,034 for software development, and UK£2,408 for educational activities. This gave a total one-off cost of initiating the system across the two register areas of UK£27,885. The additional annual cost of running the system for the two registers was UK£11,170. Based on the interviews with practice-based informants, the mean maximum annual cost per patient that the practices had to meet when using the system (including staff time and consumables) was estimated at £76.46 per patient; the minimum annual costs were zero. However, because of the semi-structured nature of the interviews, it was not possible to accurately estimate the distribution of costs within this range.

**Table 5:** Economic analysis profile (Costs expressed in 2002/03 UK£).

**Type of service/resource **	**Mean (SD) per patient**		**Effect of intervention adjusted for a difference between registers**	
	**Control**	**Intervention**	**p-value**	**Mean (95% CI)**

**NHS Costs**				
Primary care visits/consultations (n = 965)	135.61 (43.40)	136.67 (40.40)	0.96	0.50 (-21.5; 22.5)
Secondary care visits/consultations (n = 1091)	189.03 (55.40)	186.45 (68.73)	0.62	-7.41 (-37.58; 22.77)
All tests/investigations (n = 1046)	65.71 (26.28)	72.06 (28.05)	0.68	2.75 (-10.77; 16.28)
NHS pre-booked transport service (n = 1259)	19.34 (33.04)	17 (44.78)	0.49	-7.24 (-28.34; 13.85)
All drugs except insulin (n = 1330)	22.07(6.46)	20.81(6.68)	0.72	-0.55 (-3.6; 2.49)
Insulin (n = 1388)	6.13 (3.72)	6.18 (4.38)	0.83	0.20 (-1.65; 2.06)
Cardiovascular drugs (all categories) (n = 1341)	18.3 (5.38)	17.05(5.25)	0.60	-0.66 (-3.15; 1.84)
**Private costs/time use**				
All private special items/equipment* (n = 1285)	20.80 (11.05)	26.98 (12.13)	0.10	4.89 (-0.97; 10.75)
All private consultations(n = 1348)	3.21 (3.92)	2.45 (2.56)	0.49	-0.60 (-2.32; 1.12)
Costs-All private modes of transport (n = 1240)	7.43 (4.97)	6.86 (6.02)	0.47	-0.10 (-3.77; 1.78)
Patient-Pay loss because of time off (n = 1295)	1.10 (2.64)	3.73 (7.59)	0.06	3.01 (-0.15; 6.16)
Patient-Pay loss because of sick leave (n = 1195)	4.12 (12.33)	36.76 (103.08)	0.12	27.67 (-7.28; 62.63)
Patient-Hours off other activities (n = 1120)	1.67 (1.87)	0.86 (0.98)	0.07	-0.77 (-1.6; 0.07)
Patient-Days off other activities (n = 1034)	0.18 (0.29)	0.20 (0.34)	0.77	2.488E-02 (-0.15; 0.19)
Companion-Pay loss (n = 1233)	1.66 (6.62)	2.89 (9.08)	0.65	0.85 (-2.96; 4.67)
Companion-Days off (n = 734)	0.62 (0.86)	0.82 (1.11)	0.66	0.10 (-0.37; 0.58)
Companion – Hours off (n = 858)	2.50 (3.48)	2.11 (1.90)	0.74	-0.23 (-1.65; 1.19)

* Special items/equipment include: spectacles, special shoes, glucose tablets, monitoring equipment, books or videos.

## Discussion

We have evaluated an area-wide computerised diabetes register incorporating a full structured recall and individualised patient management system – one of the largest trials of its kind in terms of the number of provider units, and the largest in terms of patient numbers. The intervention produced improvements in patient attendance, improvement in four of the nine measured areas of provider adherence to recommended care (the recording of foot examination, dietary advice, blood pressure, and smoking status), and improvement in one measure of clinical control (serum cholesterol). These benefits incurred costs.

Although we showed significant improvements in the recording of drugs in the database, there were problems with the dating of the drug data. The patient reported data on drug use showed no significant differences in usage between the two groups. Given this discrepancy and the potential for inaccuracy in both data sets, the impact of the intervention on prescribing has to be regarded as unclear.

In our study which utilised provider reminders and audit and feedback within an information system, we found changes in provider adherence that were considerably larger than those identified in the review by Shojania et al., and four of our nine were statistically significant improvements [5]. However, because our data was coming from a routine register it is important to consider the possibility that, for the provider adherence variables, some of the effect was due to a recording phenomenon, and the same actions were being performed as frequently in control practices but were just not being recorded. Setting aside the fact that the recording of care, particularly in chronic disease management, is a central part of good care [24], all of the provider adherence and all of the clinical variables showed a direction of effect in favour of the intervention. In addition, four of the provider adherence variables (recording of HbA1c, cholesterol, serum creatinine, and urinary albumin:creatinine ratio) were not reliant on recording within general practices; they were routinely transferred into the diabetes databases directly from the laboratory information systems, and so would not be subject to any recording effect. Whilst these were not statistically significantly different between intervention and control groups, many clinicians would regard changes of this size as clinically significant (16% increase in HbA1c recording and 21% increase in cholesterol recording).

There is some suggestion of under-recording of data on the registers, with an apparently low proportion of people on aspirin and insulin (mirrored by an apparently high proportion of people on diet alone). This is almost certainly due to a combination of factors of which a degree of under-recording is only one. Low aspirin prescription rates could be due to patients buying aspirin directly from pharmacies rather than receiving it via prescription (common in the UK). This is supported by the figures for self-reported aspirin use being higher than those on the registers. Excluded from the study were people being treated for their diabetes solely by hospital, who are more likely to be treated on insulin and less likely to be on diet alone. However, while we have the same rates for patients treated with diet alone from the register and from self-report, self-report of insulin use was considerably higher than on the registers. This suggests that insulin use was under-recorded on the registers, but equally so for both intervention and control groups.

Unlike the studies in the review, we found no significant effect on levels of HbA1c. This may reflect the overall levels of control in our study population with baseline HbA1c of 7.7, and both groups improving to 7.3. The studies in the review were conducted in more poorly controlled populations, with median baseline HbA1c values of over 8 (and in one case over 10). Our findings also may reflect the relatively short period for which the intervention ran as fully intended. While the intervention was in place for the planned 15 months, the full intervention ran for only 9 months, and the intended patient intervention was never fully operational.

We did, however, show a modest and statistically significant lowering of serum cholesterol of 0.15 mmol/l in the intervention group compared to the control group. As the impact of the intervention on medication, including lipid-lowering therapy, was unclear from the register-derived data and negative from the patient-reported data, it is possible that this effect may be due to the increased delivery of dietary advice – one of the four areas of improvement in provider adherence to recommended care.

We showed no significant difference in patient-reported outcomes between intervention and control groups. The observed clustering in the outcome scores was smaller than that assumed in the sample size calculation, and, as we achieved the desired sample size, the lack of significant changes in patient outcomes is unlikely to be due to a lack of power. However, we do have to consider the possibility of non-response bias for all the self-reported data with a response rate of 51%, even though there was no difference between intervention and control group response rates, or on sociodemographic variables.

It is very unusual for implementation trials to include a rigorous economic evaluation [25]. Given that implementation trials do not produce a single estimate of overall effect, we have expressed the economic evaluation in terms of the profile of incurred costs. Our assessment of costs incurred by the practices was limited, and so we have only suggested a hypothetical illustration of the likely costs for an average Primary Care Trust as shown in Table 6. Whilst we could not precisely define the distribution of the costs for general practices, assuming an average cost of 25% of the range shows that the practice incurred costs would still be the single largest cost element incurred by introducing a system such as this. Whilst for any individual practice the figures would be proportionately lower, in a demand-led system such as UK general practice, coping with such innovations should be accompanied by commensurate resources. This is particularly important when, as in this case, an innovation can reside in specialist services or hospital care that has no responsibility for expenditure incurred in family or general practice. In any future study, a more detailed costing study in general practice would be important.

**Table 6:** Hypothetical example of the estimated costs of the intervention applied to an average PCT (Costs expressed in 2002/03 UK£).

	Estimated costs	Estimated costs for an average PCT^1^
1. Adapting the guidelines	£11443	£11443
2. Developing/modifying the software	£14034	£14034
3. Local educational meetings	£1204	£1204
4. Register running costs	£5585	£5,585/year
5. General practice running costs^2^	£19.11/patient/year	£72,236/year

1 Average PCT: 40 general practices, practice size 3.5 FTE doctors, list size 1800/doctor, prevalence of type 2 diabetes 1.5%. This gives 3780 patients.

2. Average cost incurred by practices assumed to be 25% of the range of £0.00 to £76.52. Includes staff time and consumables.

## Conclusion

This study has shown benefits from an area-wide, computerised diabetes register incorporating a full structured recall and individualised patient management system. However, these benefits were achieved at a cost. In future, these costs may fall as electronic data exchange becomes a reliable reality. However, as performance steadily rises it will become ever more difficult to demonstrate smaller and smaller incremental improvements. Considering our findings alongside those of Shojania's review, such developments should only be evaluated in large-scale, randomised controlled trials incorporating a full economic evaluation.

## Declaration of competing interests

DM was senior partner of Westman Medical Software, who developed the software. The company was taken over by ProWellness UK Ltd, who continue to maintain the software used in this study. The remaining authors declare that they have no competing interests.

## Authors' contributions

The study was conceived by ME, GH and JG. It was designed by ME, GH, PW, JG, NS, AV, DM and LW. It was run by PW, CS, GH, AV and ME. DM developed and modified the software. Senior clinicians and diabetes register staff in the two sites ran and maintained the intervention. NS supervised the analysis. AV conducted the economic evaluation. All authors commented on successive drafts of the paper. ME is the guarantor of the paper.

## Supplementary Material

Additional File 1 Example of a structured management sheet. The file provides an anonymised example of a structured management sheet from the enhanced diabetes register. Click here for file

Additional File 2 Table 2 (expanded). Unadjusted and adjusted register-derived process and clinical outcome data results for intervention and control groups. This table reproduces the data provided in Table 2 and also includes analyses allowing for baseline data only and register effect only. Click here for file
