# Supplementary material for: A pragmatic cluster randomised controlled trial of a Diabetes REcall And Management system: the DREAM trial
Source: Implement Sci. 2007 Feb 16;2:6. doi: 10.1186/1748-5908-2-6 (PMC1804280; doi:10.1186/1748-5908-2-6)
Supplement: Additional File 1 — Example of a structured management sheet. The file provides an anonymised example of a structured management sheet from the enhanced diabetes register. [file 1748-5908-2-6-S1.pdf]

Assessment of Previous Metabolic Data

	Previous	Latest	Management Prompt
	07/02/2001	25/02/2002	Current relevant Rx : Metformin. Add Gliclazide 80mg once daily, then 80mg twice daily, then 160mg twice daily
HbA1c	7.7	7.7	
	07/02/2001	25/02/2002	Current relevant Rx : Simvastatin. Increase statin to full dose and consider referral to a diabetes or lipid clinic.
Cholesterol	4.5	5.7	
HDL-cholesterol		1.31	
Triglyceride	2.0	3.4	
LDL-cholesterol		2.85	
	25/02/2002	05/05/2002	
S.creatinine	80	76	
	25/02/2002	26/03/2002	
U.albumin			
U.alb/creat.ratio	3.4	1.2	

Blood/Urine tests to be taken this review :

HbA1c: [x] Lipids: [x] S.creatinine: [x] U.albumin/creat.ratio: [x] U.protein output: [ ]

Assessment of Previous Clinical Examinations

	Previous	Latest	Management Prompt
	02/08/2001	01/11/2001	Discuss diet with patient or consider referral to dietitian.
BMI	33.1	33.1	
	01/11/2001	06/02/2002	If confirmed BP>140/80 mm Hg & normoalbuminuria, treat BP to reach target of 140/80. Consider ACE inhibitor &/or beta blocker as first 2 drugs of choice depending on pt's characteristics. Add other drugs, according to pts individual factors: thiazides, beta blockers, ACE Is & calcium channel blockers all effective in lowering BP. Monitor renal function at outset, within 1 wk of starting treatment & after each dose change.
Systolic BP	150	179	
Diastolic BP	80	92	
	13/12/2000	25/02/2002	
Eye exam.	ret.camera	ret.camera	
VA (Right)	6/4	6/5	
VA (Left)	6/5	6/6	
		13/12/2000	No foot exam recorded in last 14 months. Review all patients annually. Check pulses and sensation (monofilament to 10g level). For patients with no clinical evidence of neuropathy/PVD, provide general foot care education. Refer: "at risk feet" to Podiatry Clinic; absent pulses/signif.symptoms PVD to vascular surgeons; ulcers/infection URGENTLY to diabetologist or diabetic podiatrist; gangrene as EMERGENCY.
Feet exam.		clinician	
	02/02/2000	06/02/2002	Patient is obese. Discuss diet with patient or consider referral to dietitian.
Diet assess.	dietitian	clinician	
	06/02/2002	25/02/2002	Ask about smoking status. If patient smoking, consider referral to local smoking cessation guidelines.
Smoking status			
Chest pain			Ask patient if they have had any chest pain or new breathlessness. If yes, refer to local CHD guidelines if available.

Actions Taken This Clinical Review

Date	Height	Weight	BMI	BP	Qual.	Hyper	Hypos.	No.with	Fundoscopy				Visual acuity		Diet		Feet		DSN
	metres	Kg		mm Hg	prot.	epis/yr	sev./m	warning	Self	Opt.	Cam.	Opht.	Right	Left	Disc	Dietit.	Exam	Pod.	educ

SMOKING HISTORY

[x] Never [ ] Stopped in ( )

Number of cigarettes /day

ALCOHOL (u/wk)

Never [ ]

Current [0]

REFER TO:

[ ] Ophth [ ] Card. [ ] DSN

[ ] Vasc.Sg. [ ] Renal [ ] Diet.

[ ] Pod.

Date of vaccination	
Influenza	Pneumococ

CARDIOLOGY

Has paxent had cxest pain? [ ] Yes [ ] No

Blood/Urine tests taken this review :

HbA1c: [ ] Lipids: [ ] S.creatinine: [ ] U.albumin/creat.ratio: [ ] U.protein output: [ ]

Treatment Changes

The register currently has no medication data for this patient - please complete on the attached sheet. NB Consider aspirin. Aspirin (75mg/day) should be given routinely and continued for life in patients with diabetes and CHD (unless there are contraindications).
