# Supplementary material for: A pragmatic cluster randomised controlled trial of a Diabetes REcall And Management system: the DREAM trial
Source: Implement Sci. 2007 Feb 16;2:6. doi: 10.1186/1748-5908-2-6 (PMC1804280; doi:10.1186/1748-5908-2-6)
Supplement: Additional File 2 — Table 2 (expanded). Unadjusted and adjusted register-derived process and clinical outcome data results for intervention and control groups. This table reproduces the data provided in Table 2 and also includes analyses allowing for baseline data only and register effect only. [file 1748-5908-2-6-S2.doc]

| **Measure of process** | **Raw data: Summary statistics for all patients** | | | | **Estimated effect of intervention from alternative models** | | | |
| --- | --- | --- | --- | --- | --- | --- | --- | --- |
|  |  |  |  |  | **Including baseline as a covariate?** | | | |
|  |  |  |  |  | **No** | | **Yes^1^** | |
|  | **Control Practices** | | **Intervention Practices** | | **Including a difference between registers?** | | **Including a difference between registers?** | |
|  | **Baseline** | **Follow-up** | **Baseline** | **Follow-up** | **No** | **Yes** | **No^1^** | **Yes** |
| **Attendance** |  |  |  |  | **Odds ratio (95% CI)** | | | |
| Proportion of patients with at least one appointment | 73.4% | 67.7% | 74.3% | 81.7% | 1.80^†^ (0.91, 3.58) | 1.89^†^ (0.96, 3.69) | 1.97^†^ (0.99, 3.94) | 2.00^*^ (1.02, 3.91) |
| Mean number of appointments | 1.23 | 1.35 | 1.29 | 2.02 | RR = 1.34 (0.83, 2.15) | RR = 1.27 (0.88, 1.84) | RR = 1.31 (0.82, 2.09) | RR = 1.26 (0.87, 1.81) |
| **Process of care** |  |  |  |  |  |  |  |  |
| Fundoscopy recorded | 49.5% | 50.5% | 43.1% | 60.6% | 1.36 (0.81, 2.29) | 1.37 (0.82, 2.30) | 1.44 (0.87, 2.39) | 1.45 (0.88, 2.40) |
| Feet examination recorded | 46.1% | 48.8% | 48.0% | 67.3% | 1.87^*^ (1.09, 3.21) | 1.87^*^ (1.09, 3.21) | 1.87^*^  (1.09, 3.20) | 1.87^*^ (1.09, 3.21) |
| Dietary advice recorded | 19.9% | 29.2% | 25.3% | 46.3% | 2.45^*^ (1.08, 5.54) | 2.65^*^ (1.17, 6.04) | 2.05^†^ (0.95, 4.41) | 2.77^*^ (1.22, 6.29) |
| Smoking status recorded | 34.2% | 48.0% | 36.9% | 66.0% | 1.93^†^ (0.99, 3.79) | 1.93^†^ (0.99, 3.77) | 2.36^*^ (1.15, 4.86) | 2.43^*^ (1.18, 5.00) |
| Was subject a smoker? | 19.3% | 19.6% | 20.7% | 21.4% | 1.10 (0.76, 1.61) | 1.10 (0.74, 1.64) | 0.74 (0.36, 1.51) | 0.72 (0.38, 1.37) |
| BP recorded | 59.3% | 48.3% | 55.3% | 71.4% | 2.28^*^ (1.14, 4.59) | 2.28^*^ (1.13, 4.59) | 2.19^*^ (1.08, 4.45) | 2.14^*^ (1.06, 4.36) |
| HbA1c recorded? | 64.0% | 66.0% | 60.9% | 79.0% | 1.39 (0.67, 2.87) | 1.56 (0.79, 3.08) | 1.49 (0.72, 3.06) | 1.58 (0.81, 3.08) |
| Cholesterol recorded | 57.0% | 61.1% | 53.3% | 78.0% | 1.42 (0.70, 2.88) | 1.64 (0.85, 3.17) | 1.50 (0.77, 2.93) | 1.66 (0.89, 3.12) |
| Creatinine recorded | 48.0% | 60.4% | 53.0% | 73.4% | 1.20 (0.58, 2.52) | 1.38 (0.70, 2.74) | 1.35 (0.66, 2.75) | 1.36 (0.72, 2.52) |
| Albumen:creatinine ratio recorded | 26.8% | 29.7% | 30.2% | 40.4% | 1.24 (0.60, 2.56) | 1.53^†^ (0.92, 2.53) | 1.14 (0.59, 2.21) | 1.60^†^ (0.98, 2.60) |
| **Clinical** |  |  |  |  | **Mean difference (95% CI)** | | | |
| Mean most recent systolic blood pressure | 144.5 | 144.6 | 145.8 | 144.2 | -0.52 (-3.86, 2.82) | -0.72 (-4.13, 2.68) | -1.45 (-4.40, 1.48) | -1.56 (-4.54, 1.42) |
| Mean most recent diastolic blood pressure | 80.2 | 78.1 | 79.2 | 77.8 | 0.22 (-1.31, 1.76) | 0.18 (-1.39, 1.75) | -0.17 (-1.63, 1.29) | -0.40 (-1.78, 0.97) |
| Mean most recent HbA1c | 7.56 | 7.35 | 7.75 | 7.32 | 0.01 (-0.28, 0.30) | -0.01 (-0.19, 0.17) | -0.02 (-0.27, 0.22) | -0.04 (-0.18, 0.10) |
| Mean most recent cholesterol | 5.27 | 5.06 | 5.23 | 4.94 | -0.22^***^ (-0.35, -0.09) | ‑0.22^***^ (-0.34, -0.09) | -0.16^**^ (-0.26, -0.05) | -0.15^**^ (-0.25, -0.06) |
| Mean most recent creatinine | 93.1 | 96.1 | 91.8 | 95.7 | -0.62 (-3.33, 2.10) | -0.61 (-3.33, 2.10) | 0.15 (-1.40, 1.70) | 0.21 (-1.27, 1.70) |
| Mean most recent albumen creatinine ratio | 8.99 | 8.45 | 8.48 | 8.05 | -0.4 (-3.7, 3.0) | -0.4 (-3.8, 3.0) | -1.9 (-4.7, +0.9) | -1.6 (-4.4, 1.2) |
| **Diabetes medication** |  |  |  |  | **Relative risk (95% CI)** | | | |
| Tablets (Biguanide or Sulphonylurea or Thiazols) | 944 (49.0%) | 646 (38.9%) | 1128 (58.5%) | 923 (55.5%) | 0.99 (0.83, 1.19) | 1.01 (0.87, 1.17) | 1.06 (0.93, 1.20) | 1.06 (0.94, 1.19) |
| Metformin (Biguanide) | 424 (22.0%) | 343 (20.6%) | 573 (29.7%) | 530 (31.9) | 1.14 (0.78, 1.66) | 1.14 (0.78, 1.67) | 1.08 (0.82, 1.44) | 1.07 (0.81, 1.41) |
| Insulin | 57 (3.0%) | 54 (3.2%) | 75 (3.9%) | 75 (4.5%) | 1.12 (0.73, 1.71) | 1.11 (0.72, 1.70) | 1.14 (0.83, 1.57) | 1.15 (0.83, 1.58) |
| **Cardiovascular risk factor drugs** |  |  |  |  |  |  |  |  |
| Aspirin | 10 (0.5%) | 34 (2.0%) | 164 (8.5%) | 308 (18.5%) | 2.26* (1.08, 4.71) | 2.47* (1.15, 5.32) | 1.94 (0.91, 4.08) | 2.08* (1.00, 4.32) |
| Ace Inhibitor | 17 (0.9%) | 31 (1.9%) | 103 (5.3%) | 185 (11.1%) | 2.12* (1.10, 4.11) | 2.15* (1.10, 4.18) | 1.96* (1.03, 3.74) | 2.03* (1.08, 3.78) |
| Ace Inhibitor or Angiotensin-II receptor antagonist | 21 (1.1%) | 38 (2.3%) | 109 (5.7%) | 192 (11.6%) | 2.10* (1.12, 3.94) | 2.12* (1.12, 3.99) | 1.85* (1.00, 3.43) | 1.86* (1.03, 3.38) |
| Antihypertensive | 118 (6.1%) | 131 (7.9%) | 274 (14.2%) | 415 (25.0%) | 1.91* (1.10, 3.33) | 2.09** (1.20, 3.64) | 1.81* (1.12, 2.94) | 1.89* (1.16, 3.08) |
| Lipid lowering | 110 (5.7%) | 79 (4.8%) | 290 (15.0%) | 418 (25.2%) | 1.60 (0.88, 2.90) | 1.57 (0.87, 2.84) | 1.76* (1.04, 2.97) | 1.66 (0.99, 2.79) |
| **Any medication** | 1674 (86.9%) | 1283 (77.2%) | 1838 (95.4%) | 1549 (93.2%) | 0.98 (0.91,1.05) | 0.99 (0.92, 1.06) | 1.05 (0.95, 1.15) | 1.01 (0.94, 1.08) |

^1^ This model corresponds to the pre-specified analysis

^†^ p < 0.1

* p < 0.05

** p < 0.01

*** p < 0.001
